# Effect of intracranial pressure on photoplethysmographic waveform in different cerebral perfusion territories: A computational study

**DOI:** 10.3389/fphys.2023.1085871

**Published:** 2023-03-16

**Authors:** Haipeng Liu, Fan Pan, Xinyue Lei, Jiyuan Hui, Ru Gong, Junfeng Feng, Dingchang Zheng

**Affiliations:** ^1^ Research Centre for Intelligent Healthcare, Coventry University, Coventry, United Kingdom; ^2^ College of Electronics and Information Engineering, Sichuan University, Chengdu, China; ^3^ Brain Injury Center, Renji Hospital, School of Medicine, Shanghai Jiao Tong University, Shanghai, China

**Keywords:** intracranial pressure (ICP), photoplethysmography (PPG), windkessel effect, computational simulation, artery network, cerebral microcirculation

## Abstract

**Background:** Intracranial photoplethysmography (PPG) signals can be measured from extracranial sites using wearable sensors and may enable long-term non-invasive monitoring of intracranial pressure (ICP). However, it is still unknown if ICP changes can lead to waveform changes in intracranial PPG signals.

**Aim:** To investigate the effect of ICP changes on the waveform of intracranial PPG signals of different cerebral perfusion territories.

**Methods:** Based on lump-parameter Windkessel models, we developed a computational model consisting three interactive parts: cardiocerebral artery network, ICP model, and PPG model. We simulated ICP and PPG signals of three perfusion territories [anterior, middle, and posterior cerebral arteries (ACA, MCA, and PCA), all left side] in three ages (20, 40, and 60 years) and four intracranial capacitance conditions (normal, 20% decrease, 50% decrease, and 75% decrease). We calculated following PPG waveform features: maximum, minimum, mean, amplitude, min-to-max time, pulsatility index (PI), resistive index (RI), and max-to-mean ratio (MMR).

**Results:** The simulated mean ICPs in normal condition were in the normal range (8.87–11.35 mm Hg), with larger PPG fluctuations in older subject and ACA/PCA territories. When intracranial capacitance decreased, the mean ICP increased above normal threshold (>20 mm Hg), with significant decreases in maximum, minimum, and mean; a minor decrease in amplitude; and no consistent change in min-to-max time, PI, RI, or MMR (maximal relative difference less than 2%) for PPG signals of all perfusion territories. There were significant effects of age and territory on all waveform features except age on mean.

**Conclusion:** ICP values could significantly change the value-relevant (maximum, minimum, and amplitude) waveform features of PPG signals measured from different cerebral perfusion territories, with negligible effect on shape-relevant features (min-to-max time, PI, RI, and MMR). Age and measurement site could also significantly influence intracranial PPG waveform.

## 1 Introduction

Intracranial pressure (ICP), defined as the pressure within the craniospinal compartment, is an important physiological parameter that reflects the biomechanical status of the brain. ICP is derived from cerebral blood and cerebrospinal fluid (CSF) circulatory dynamics. ICP can be significantly changed in many neurological diseases (Czosnyka and Pickard, 2004). For decades, ICP monitoring has been a cornerstone of traumatic brain injury (TBI) management (Stocchetti et al., 2014). Currently, external ventricular drain (EVD) is considered as the gold standard of ICP monitoring due to its accuracy with additional function of CSF drainage (Harary et al., 2018). In EVD measurement, the ICP is transmitted into an external saline-filled tube through a strain-gauge transducer for pressure measurement. The insertion of the tube is invasive with a 5%–7% risk of hemorrhage, and is difficult to perform in some patients with inherently small ventricles size (Harary et al., 2018). To ease the postoperative ICP monitoring especially in TBI patients, it is essential to develop non-invasive methods of ICP monitoring.

The photoplethysmography (PPG) technology has been applied in the daily monitoring of many physiological parameters and may enable non-invasive long-term ICP monitoring. The cyclic fluctuations of a PPG signal reflect volumetric changes in the microcirculation, which is regulated by many physiological factors, e.g., respiratory pattern, arterial stiffness, and the mechanical properties of surrounding tissues. Therefore, PPG signals derived from the distal area of intracranial arteries might reflect ICP-related changes in cerebral microcirculation. The infra-red PPG signals measured from extracranial skin surface could reflect the intracranial microcirculation in different cerebral perfusion territories (Viola et al., 2013). A recent pilot study showed that the PPG signal recorded non-invasively from forehead can detect apnea-induced cerebral blood flow oscillations (Alex et al., 2019). In a pilot study on 14 subjects, Morgan et al. (2021) estimated ICP using retinal vein PPG signal and achieved clinically acceptable accuracy (−0.35 ± 3.6 mmHg). These studies indicated that intracranial PPG signals measured from extracranial areas might be a promising tool for non-invasive ICP monitoring. However, it is uncertain if ICP changes could generate waveform changes of intracranial PPG signals, with a lack of theoretical basis and in-depth analysis from a physiological perspective.

Computational modelling and simulation based on biomechanical and hemodynamic theories have been widely applied in the investigation of intracranial blood flow and ICP (Liu et al., 2020b). Especially, the Windkessel model is a highly simplified one where the resistance and compliance in the circulatory system are simulated as resistors and capacitors in a circuit (Alastruey et al., 2007). The unidirectional flow in the CSF circulation can be simulated using diode elements (Ursino and Di Giammarco, 1991). Recently, data-driven algorithms were proposed to improve the accuracy of ICP simulation. It was suggested that ICP can be computationally estimated from the cerebral blood flow and blood pressure (Kashif et al., 2012). However, the biomechanical properties of arteries are non-linear and age-dependent, which was not fully considered in existing models of ICP simulation. Moreover, the hemodynamic data in existing models were from invasive measurement. The relationship between non-invasively measured intracranial PPG and ICP has not been comprehensively investigated using computational modelling.

**FIGURE 1:**
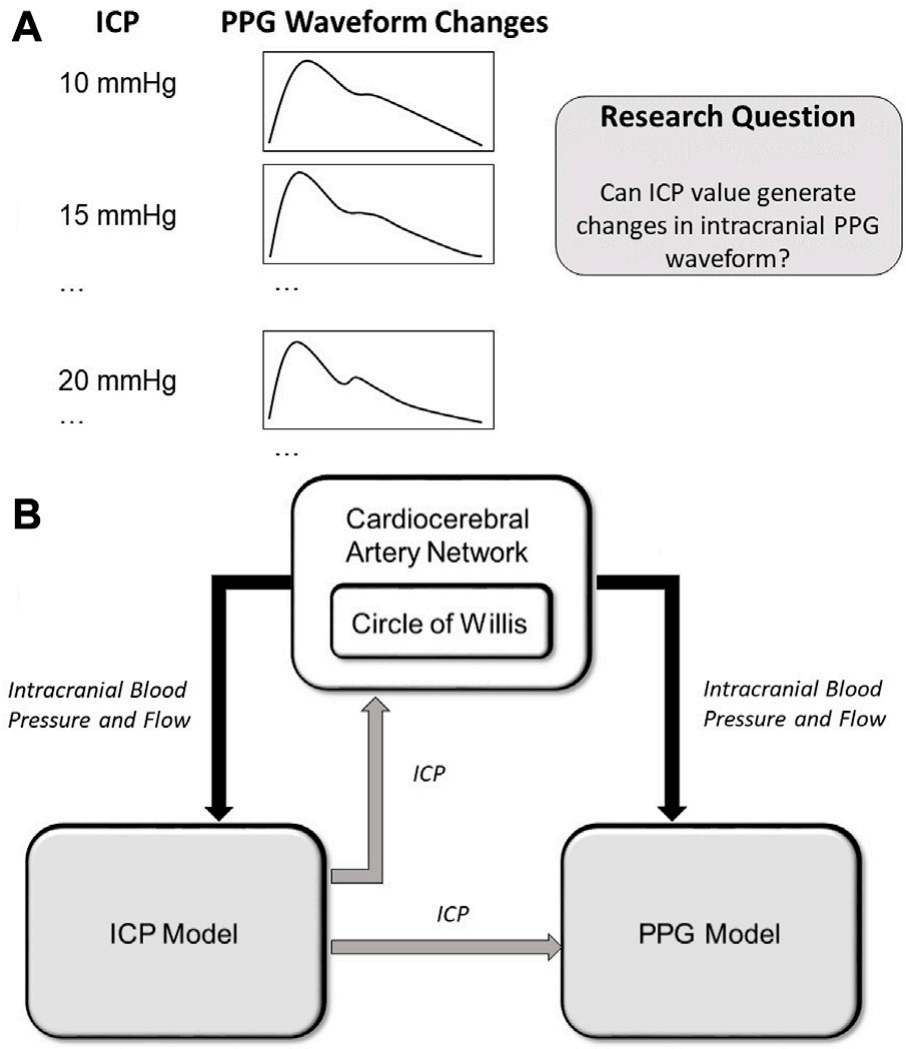
**(A)** Scientific hypothesis of this paper: changes in ICP values can lead to PPG waveform changes which can be computationally simulated. **(B)** Structure of the computational model. The arrows show the data flows. ICP: intracranial pressure; PPG: photoplethysmography.

To fill this research gap, we aim to develop a computational model of intracranial PPG signals and investigate if the changes in ICP could lead to the changes in intracranial PPG signals of different cerebral perfusion territories (Figure 1).

## 2 Methods

### 2.1 Overview of the computational model

As shown in Figure 1A, we hypothesize that the changes in ICP can lead to waveform changes in intracranial PPG signals. To verify this hypothesis, we developed a computational model to simulate the PPG signals of different cerebral perfusion territories in different ICP conditions. The computational model consists of three parts: A cardiocerebral artery network, an ICP model, and a PPG model (Figure 1B). The cardiocerebral artery network simulated the blood flow of intracranial arteries and the local blood pressure, which were transmitted to the ICP and PPG models as model input. The ICP signal derived from the ICP model was transmitted back to the cardiocerebral artery networks to generate the boundary conditions. At the same time, the ICP model generates the input of the PPG model at microcirculatory level. In summary, the three parts are interactive. All the components of the three parts are based on lump-parameter Windkessel models. The computational models are detailed in the following subsections. The parameters in the models are listed in Table 1.

**TABLE 1:** Data sources of the parameters in the computational models.

Models	Data sources and references of the parameters
Cardiocerebral artery network	Anatomic parameters of arteries: Table 1 of (Alastruey et al., 2007); Calculation of parameters of Windkessel elements: Eqs 1–3 and Table 1 of (Zhang et al., 2014)
Age-dependent non-linear arterial capacitance	Parameters in age-dependent capacitance of aorta: Table 1 and Eq. 5 of (Wesseling et al., 1993); Parameters of age-dependent capacitance of CCA: basic function from Table 1 and Eq. 1 of (Kopustinskas et al., 2010), References pressure (mean pressure of healthy adults) from the subsection “Theoretical Background” and Eq. 1 of (Giudici et al., 2022), age-dependent capacitance changes from Figure 2 of (Vriz et al., 2017)
ICP model	Parameters of circuit elements in the ICP model: Table 1 of (Lee et al., 2015); Piecewise ICP function: References ICP value (5 mmHg) from (Ryding, 2017) and (Alperin et al., 2000); parameter in the inverse proportional function from the subsection “Assignment of Parameter Basal Values” of (Ursino and Lodi, 1997) and Figure 8 of (Ursino and Di Giammarco, 1991)
PPG model	Values of distal resistance and capacitance: same as those in cardiocerebral artery network; Ratios between different components: Table 1 of (Tanaka, 2022)

CCA, common carotid artery; ICP, intracranial pressure; PPG, photoplethysmography.

### 2.2 Cardiocerebral artery network

**FIGURE 2:**
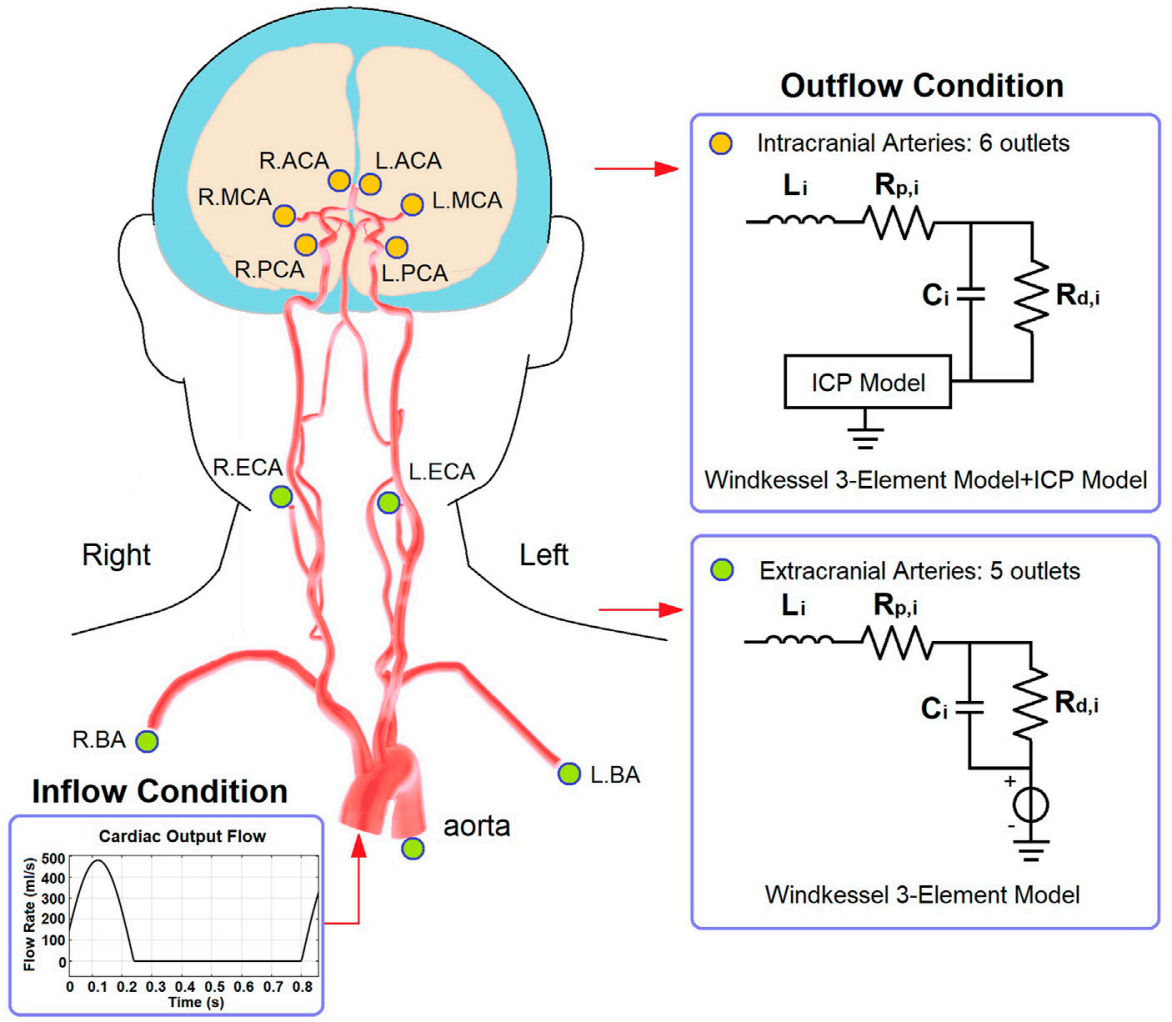
The structure of cardiocerebral artery network and the boundary conditions. The illustration of artery structure is adapted from Figure 2 of (Kang et al., 2021).

The cardiocerebral arterial network was based on the classic brain circulation model proposed by Alastruey et al. (2007), with outlet boundary conditions of intracranial arteries modified to include the effect of ICP on cerebral microcirculation. The cardiac output flow (i.e., the inflow of the aorta) was used as the inlet boundary condition. The structure of the artery network starts from the aorta and includes the major branches of intracranial arteries (Figure 2). The intermediate (i.e., connecting other artery segments without any inlet or outlet) branches included: ascending aorta, aortic arch (in two segments), brachiocephalic artery, common carotid arteries (left and right), subclavian arteries (left and right), vertebral arteries (left and right), internal carotid arteries (left and right, both in two segments), basilar artery, as well as the connecting arteries in the Circle of Willis, i.e., posterior communicating arteries (left and right), anterior communicating artery, and the first segments of anterior and posterior cerebral arteries (left and right, for both). Each intermediate artery was simulated using a three-element Windkessel model which consisted of a resistor, a capacitor, and an inductor that reflected the resistance, capacitance, and inductance of an elastic artery wall, respectively (Figure 2). The anatomic properties of the arteries and the methods of calculating the values of circuit elements can be found in Alastruey et al. (2007) and Zhang et al. (2014) (Table 1).

Regarding the outlets, the extracranial ones included thoracic aorta, brachial arteries (left and right), and external carotid arteries (left and right). These arteries were connected to a 3-element Windkessel model (Figure 2). The resistance included peripheral and distal ones which denoted the flow resistances in the artery and microcirculation, respectively. The outlet pressure was the venous pressure (5 mmHg) which was simulated by a voltage source. For the intracranial arteries (anterior, middle, and posterior cerebral arteries, left and right for all), the outlet pressure at microvascular level (i.e., prearteriole pressure) was derived from the ICP value generated by the ICP model.

### 2.3 Age-dependent non-linear arterial capacitance

To simulate the artery blood flow in different age groups, we used age-dependent parameters in the Windkessel models of aorta and common carotid arteries.

In the aorta model, we used the pressure-dependent Windkessel capacitance element proposed by Wesseling et al. (1993). The capacitance value depends non-linearly on the pressure:

$$C_{A}\left(P\right)=\frac{A_{\max}∙L}{\pi P_{1}\left[1+{\left(\frac{P-P_{0}}{P_{1}}\right)}^{2}\right]}$$ (1)

where $A_{\max}$ is the maximal cross-sectional area, approximated as 5.8 ${cm}^{2}$ for male adults, L is the length of aorta, P denotes the local blood pressure, whilst parameter $P_{0}$ and $P_{1}$ are age-dependent reference pressure values.

$$P_{0}=\left(76-0.98*age\right)\;mmHg\;age\in \left[20,70\right]$$ (2)

$$P_{1}=\left(57-0.44*age\right)\;mmHg\;age\in \left[20,70\right]$$ (3)

The biomechanical relationship between the capacitance of common carotid artery and local blood pressure is described by a non-linear exponential function:

$$C_{CCA}\left(P\right)=a∙e^{-b∙P\left(t\right)}$$ (4)

where $a=3.14\;ml*{mmHg}^{-1}$ , $b=0.018\;{mmHg}^{-1}$ , and $P\left(t\right)$ denotes transient value of blood pressure in common carotid artery which is a major source of the capacitance effect on intracranial blood flow (Kopustinskas et al., 2010).

$$C_{CCA}\left(P\right)=C_{CCA}\left(P_{ref}\right)∙e^{-b∙\left[P\left(t\right)-P_{ref}\right]}∙\left[1.3-0.012*\left(age-20\right)\right]\;age\in \left[20,70\right]$$ (5)

where $P_{ref}=100\;mmHg$ is an established value for mean pressure of healthy adults and has been used in computational simulation studies (Giudici et al., 2022). The age-dependent function is based on a large-scale physiological measurement of common carotid artery stiffness in 900 healthy subjects (Vriz et al., 2017).

### 2.4 ICP model

**FIGURE 3:**
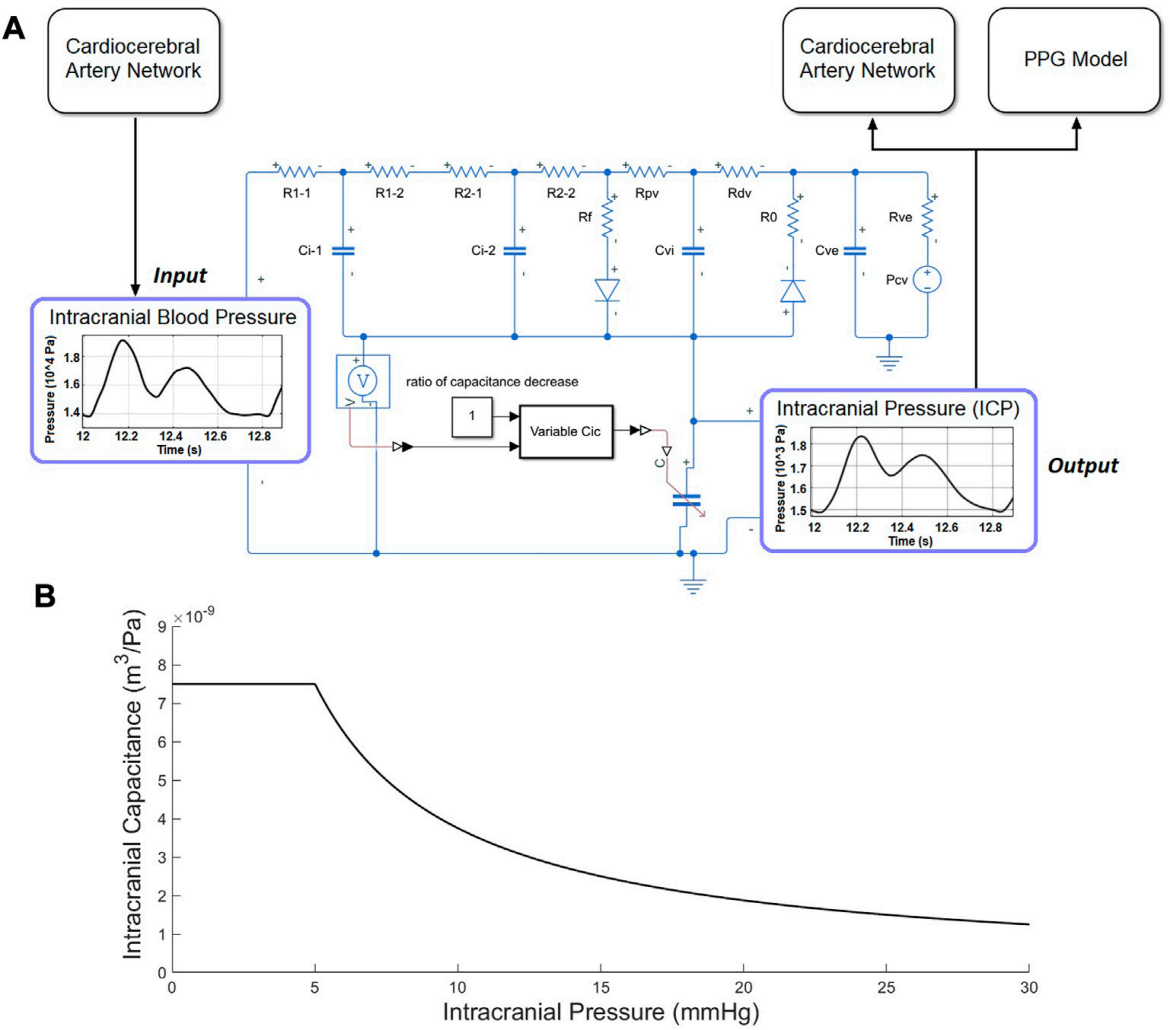
ICP model. **(A)** Electric analog of the human intracranial hydrodynamics for ICP simulation. R1-1, R1-2, and Ci-1: hydraulic resistance and compliance of the proximal arterial cerebrovascular bed (basal brain arteries and large pial arteries), respectively; R2-1, R2-2, and Ci-2: hydraulic resistance and compliance of the distal arterial cerebrovascular bed (medium and small pial arteries), respectively; Cic: intracranial tissue compliance; Cvi: intracranial venous compliance; Rpv: hydraulic resistance of the proximal venous cerebrovascular bed; Rdv: hydraulic resistance of the distal venous cerebrovascular bed (lateral lacunae and bridge veins); Rf: CSF formation resistance; Ro: CSF outflow resistance; Rve and Cue: hydraulic resistance and compliance of the extracranial venous pathways; Pcv: central venous pressure. **(B)** The piecewise function between ICP and intracranial capacitance.

The computational model for continuous ICP simulation was based on the classic model proposed by Ursino and Di Giammarco (1991) which has been widely used in ICP estimation (Lee et al., 2015). The model includes resistors and capacitors to simulate the overall resistance and capacitance of intracranial arteries, microcirculation, and veins, respectively (Figure 3). Two diodes were used to simulate the unidirectional flow in the CSF circulation.

The intracranial capacitance is a piecewise function of ICP, which is a constant when ICP<5 mmHg (venous pressure) and depends non-linearly on ICP when ICP≥5 mmHg :

$$C=\left\{\begin{array}{c} 7.502*10^{-9}*{Ratio}_{CD}\;ICP\in [0,666.5)\\ \frac{5*10^{-6}}{ICP}*{Ratio}_{CD}\;ICP\in [\;666.5,+\infty ) \end{array}\right.$$ (6)

where the unit of ICP and intracranial capacitance are Pa and $m^{3}/Pa$ , respectively. ${Ratio}_{CD}$ denotes the ratio of intracranial capacitance decrease, which is used to simulate the pathological conditions due to the brain injury with acute increase of brain tissue volume where ICP increases. The connection point of the two subintervals (5 mmHg) was modified from the reference pressure of 6 mmHg in (Ryding, 2017) to match the reference venous pressure. The reference ICP value of 5 mmHg is also in accordance with the clinical observation after the withdrawal of CSF (Alperin et al., 2000). Both normal and pathological situations were simulated, therefore, the parameter in the inverse proportional function (5*10^−6^ m^3^, or 5 ml) was set marginally below the normal range (6.66–20 ml) derive from (Ursino and Lodi, 1997) and within the range used in the simulation of pathological situations (1.92–6.41 ml) (Ursino and Di Giammarco, 1991).

### 2.5 PPG model

**FIGURE 4:**
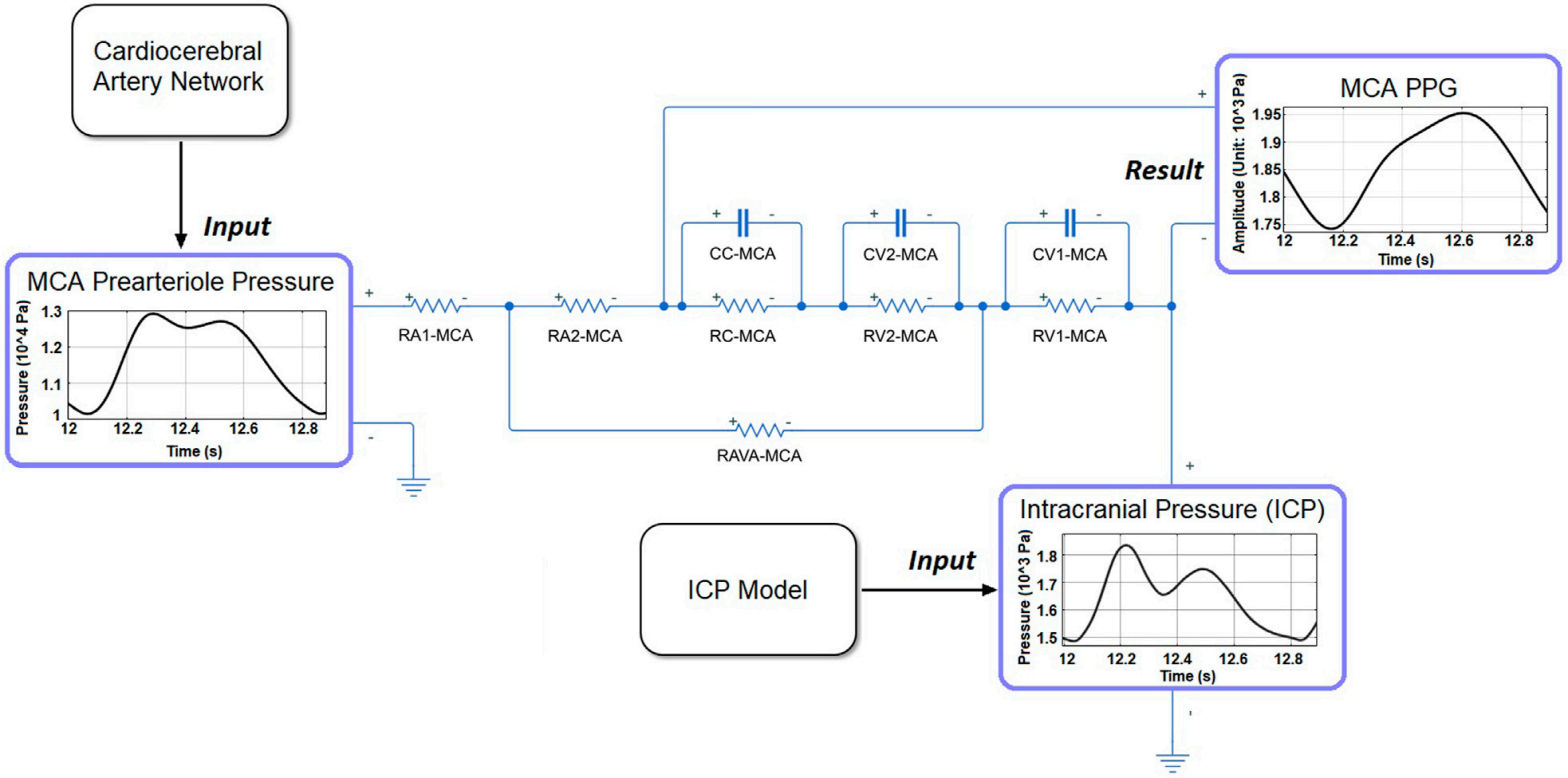
The structure of microcirculatory model to generate the PPG signal in MCA territory. RA1-MCA and RA2-MCA: equally divided arteriole resistances. RC-MCA: capillary resistance. RV2-MCA and RV1-MCA: equally divided venule resistances. CC-MCA: capillary capacitance. CV1-MCA and CV2-MCA: equally divided venular capacitances.

The PPG model was based on a cerebral microcirculation model including arteriole, capillary, and venule components (Figure 4) (Tanaka, 2022). The ratios of element values among the different components were from physiological measurement results of human cerebral circulation (Mandeville et al., 1999). The inputs of the model include prearteriole pressure and ICP generated by the cardiocerebral artery network and ICP model, respectively. The PPG signals were generated from distal perfusion territories of anterior, middle, and posterior cerebral arteries (ACA, MCA, and PCA) on the left side. For the territory of a cerebral artery (e.g., MCA), the arteriovenous anastomoses in brain tissues were simulated by a resistance between the middle points of arteriole and venule components (RAVA-MCA in Figure 4). The PPG signal was simulated as the voltage along the capacitance elements in the Windkessel model (Figure 4). Therefore, the simulation result (“simulated PPG”) reflects the pressure drop on microvascular level induced by the volumetric changes from which the PPG signal originates, whereas the unit is in Pa instead of V or mV.

### 2.6 PPG waveform features

**FIGURE 5:**
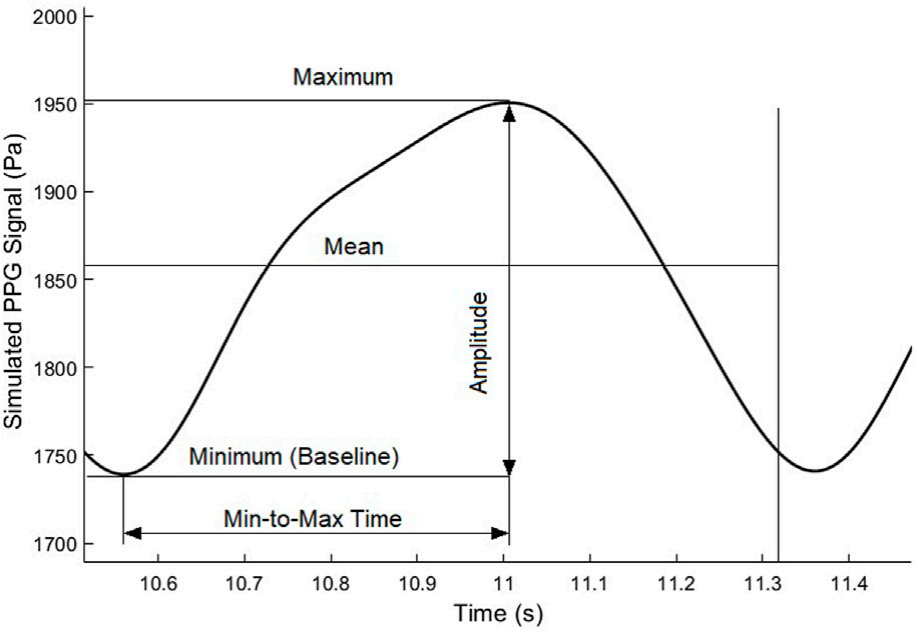
Basic waveform features of a simulated PPG signal in a cardiac cycle.

To quantitatively investigate the ICP-induced changes of PPG waveform, we used five waveform features, as shown in Figure 5. Besides the maximum and minimum (i.e., baseline) values, we calculated the mean value as the integration of the PPG signal in a cardiac cycle divided by the length of a cardiac cycle (T): $\frac{\int_{0}^{T}PPG\left(t\right)dt}{T}$ , where *PPG(t)* is the transient value of simulated PPG signal, and T=0.8s . The amplitude was defined as the difference between the maximum and the minimum: $Amplitude={PPG}_{\max}-{PPG}_{\min}$ . The min-to-max period was defined as the length of the period from minimum to maximum, which was named as rising time in existing studies on finger PPG signals where the systolic period was clearly observable (Khalid et al., 2020).

Based on the directly measured basic waveform features, we calculated three secondary waveform features which have been applied in hemodynamic research: pulsatility index (PI): $PI=\frac{{PPG}_{\max}-{PPG}_{\min}}{{PPG}_{mean}}$ ; resistive index (RI): $RI=\frac{{PPG}_{\max}-{PPG}_{\min}}{{PPG}_{\max}}$ , and the ratio between maximum and mean values of PPG signal, i.e., max-to-mean ratio (MMR): $MMR=\frac{{PPG}_{\max}}{{PPG}_{mean}}$ . The definitions of PI and RI were in accordance with those in 4D flow magnetic resonance imaging (MRI) observation of cerebral microcirculation based on flow velocity (Rivera-Rivera et al., 2015).

### 2.7 Simulation and evaluation

The simulation was performed on MATLAB-Simulink (Version: r2021a, MathWorks, Natick, MA, United States). We simulated the ICP and PPG signals in male subjects of three ages: 20, 40, and 60 years old. The simulation was repeated in four pathophysiological conditions of intracranial capacitance decrease: 0 (i.e., normal status), 25%, 50%, and 75%. To verify the model, the ICP values simulated at normal condition were compared with the results of existing physiological measurement. Each simulation lasted 30 s. To avoid any initial effect, the features were measured in the first cardiac cycle after 10 s when the signal was stable. The PPG waveform features derived were quantitatively compared between different intracranial capacitance conditions to investigate if ICP changes could lead to the waveform changes of intracranial PPG signals.

## 3 Results

### 3.1 Model validation: ICP and PPG waveforms in different ages

**FIGURE 6:**
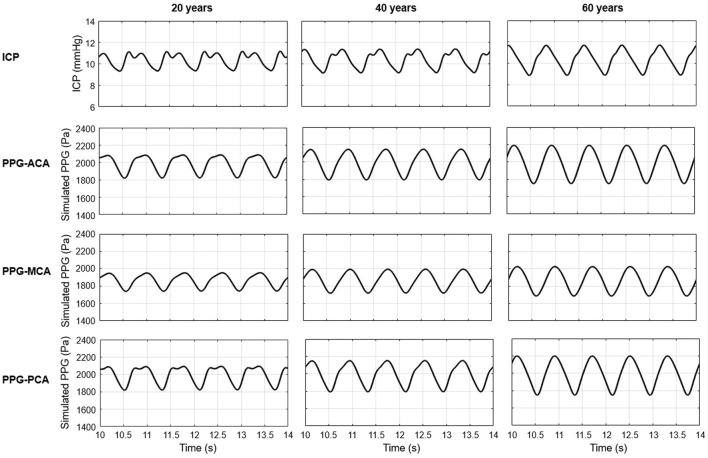
Simulated ICP and PPG waveforms during five cardiac cycles (10–14s) in 20, 40, and 60 years old healthy male subjects with normal intracranial capacitance.

**FIGURE 7:**
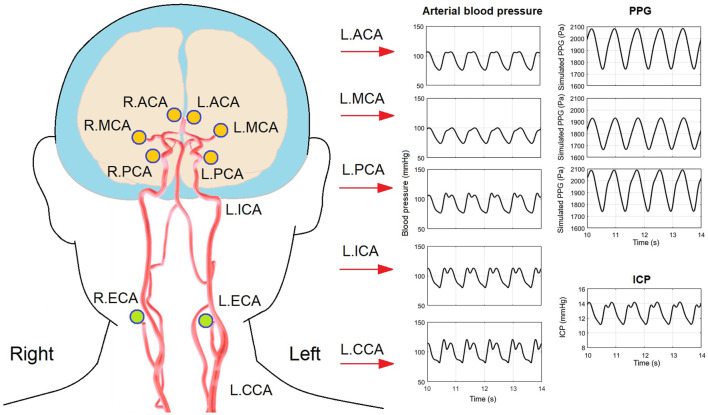
Arterial blood pressure, PPG, and ICP waveforms in a 40-years old subject with 20% decrease of intracranial capacitance. The illustration of artery structure is adapted from (Kang et al., 2021).

As shown in Figure 6, the simulated ICP signals of 20, 40, and 60 years old subjects with normal intracranial capacitance have minor differences in waveform but are similar in range: 9.31–11.12, 9.13–11.35, and 8.87–11.68 mmHg, with nearly identical mean values of 11.12, 11.35, and 11.68 mmHg (Figure 7). These mean ICP values were within the normal range of healthy adults: 10–15 mmHg (Rangel-Castillo et al., 2008).

As to the PPG waveform, it can be observed that PPG signals of ACA, MCA, and PCA territories are similar in amplitude and baseline, but different in waveform (Figure 6). There is no sharp fluctuations in the PPG waveform, which is in accordance with the fact that high-frequency components (i.e., sharp fluctuations) are absorbed by the capacitance of large arteries before arriving arterioles. The results of simulated PPG waveform features in Table 2 are in accordance with the 4D MRI flow observations that PI is large in PCA compared with MCA, and in older subjects (Rivera-Rivera et al., 2015).

**TABLE 2:** Simulated PPG waveform features in normal intracranial capacitance.

	ACA			MCA			PCA		
Age (years)	20	40	60	20	40	60	20	40	60
PI	0.134	0.178	0.222	0.114	0.147	0.182	0.137	0.181	0.226
RI	0.127	0.165	0.201	0.108	0.138	0.167	0.130	0.167	0.205
MMR	1.052	1.081	1.104	1.047	1.069	1.085	1.050	1.079	1.104

PI, pulsatility index; RI, resistive index; MMR, max-to-mean ratio.

As a more general case of all the simulations, Figure 7 shows the simulated waveforms of arterial blood pressure, PPG, and ICP of a 40 years old subject with 25% decrease of intracranial capacitance. It can be observed that the dicrotic notch and secondary peak are blurred with a flat systolic peak in the arterial blood pressure of intracranial arteries, which reflects the buffering effect of intracranial capacitance on the pulse wave (i.e., neutralization of backward wave) and is basically in accordance with existing modelling studies (Blanco et al., 2017; Schollenberger et al., 2021).

Therefore, the model can reliably simulate the ICP values in subjects with different ages, and reflect the waveform features of human cerebral microcirculation in different perfusion territories.

### 3.2 ICP values in different intracranial capacitance conditions

**FIGURE 8:**
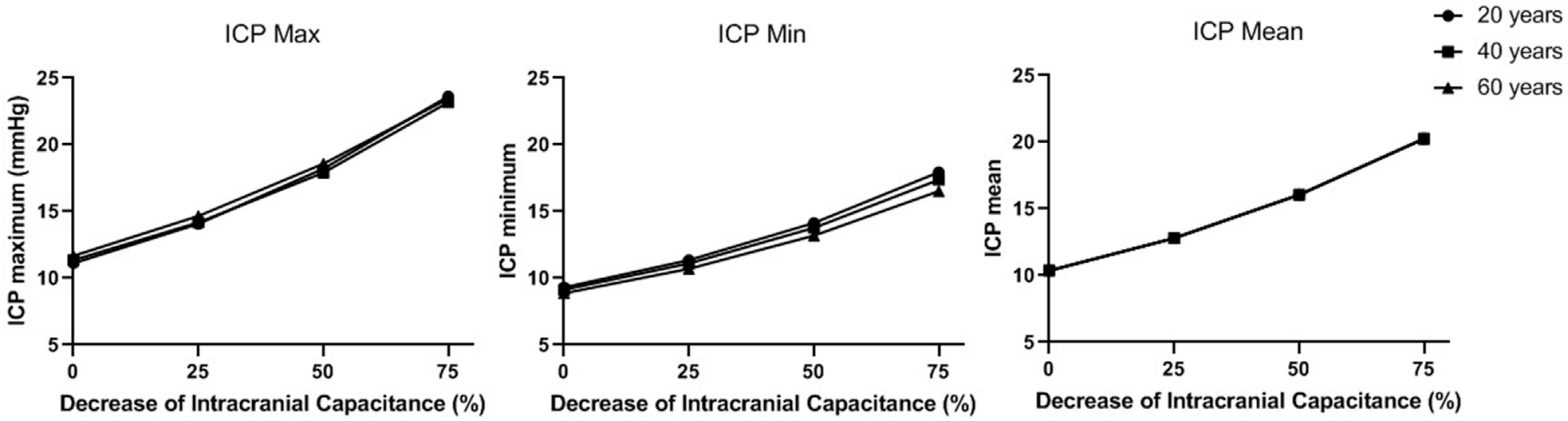
The maximum, minimum, and mean values of ICP in a cardiac cycle in different intracranial capacitance conditions.

As shown in Figure 8, ICP increases when intracranial capacitance decreases. Between different ages, the differences in maximum and minimum of ICP are very limited, while the difference in mean ICP is even negligible. With 50% decrease of intracranial capacitance, the mean values of ICP in all three ages are above 15 mmHg (16–16.1 mmHg), which is beyond the normal range (7–15 mmHg). With 75% decrease of intracranial capacitance, the mean values of ICP in all three ages are marginally beyond 20 mmHg (20.2–20.3 mmHg) where clinical intervention is recommended (Rangel-Castillo et al., 2008).

### 3.3 PPG waveform features in different intracranial capacitance conditions

**FIGURE 9:**
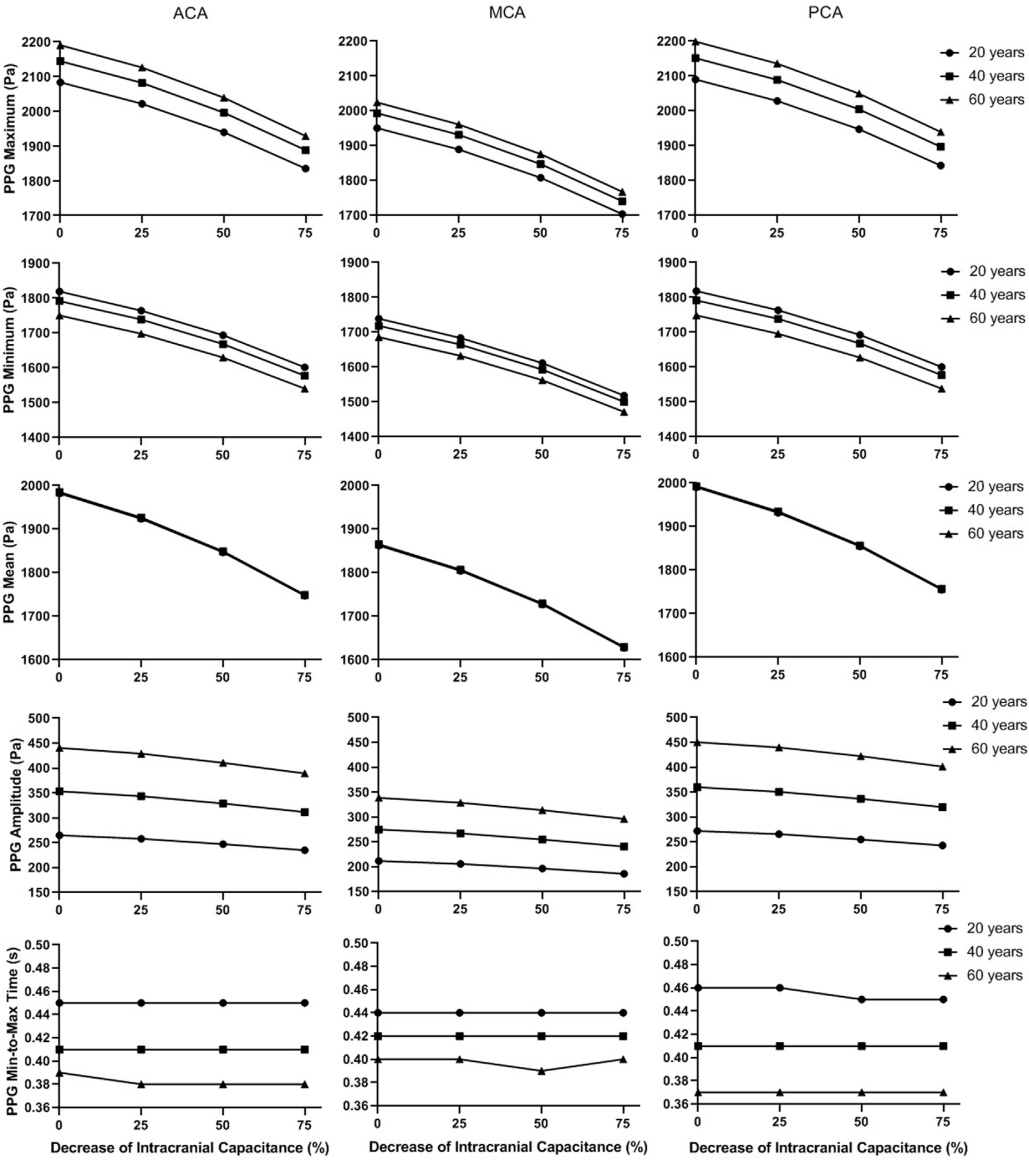
The basic waveform features of the simulated PPG signals in different intracranial capacitance conditions.

**FIGURE 10:**
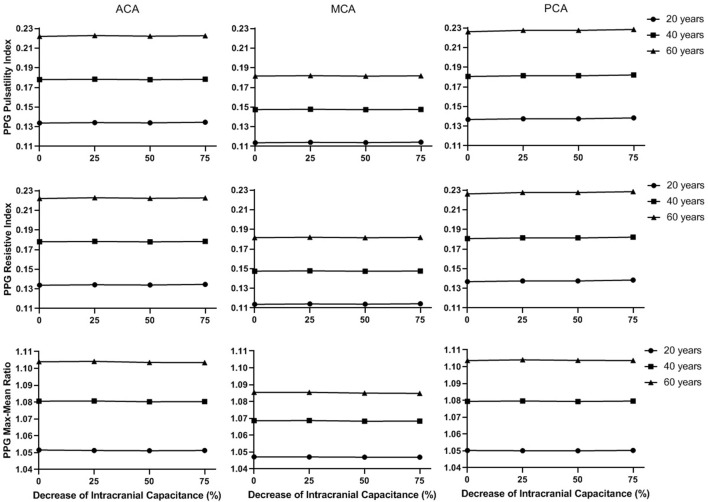
The secondary waveform features of the simulated PPG signals in different intracranial capacitance conditions.

Figure 9 and Figure 10 illustrate the effects of age and intracranial capacitance condition on the basic and secondary PPG features in different cerebral perfusion territories.

In Figure 9, the maximum, minimum, and mean values significantly decrease with intracranial capacitance, while the ICP increases from <11.5 mmHg to hypertensive condition (>20 mmHg). Meanwhile, there is a minor decrease in amplitude. In contrast, there is no consistent changes in min-to-max time or any secondary waveform feature (Figure 10) where the maximal relative difference is less than 2% among all intracranial capacitance conditions.

On the other hand, we observed significant effects of age and cerebral perfusion territory on all the waveform features. When age increases, maximum, amplitude, PI, RI, and MMR are higher, while the minimum and min-to-max time are lower, with negligible changes of the mean. Compared with PCA and ACA territories, MCA territory has lower maximum, minimum, amplitude, PI, RI, and MMR, with lower age-relevant differences in min-to-max time (Figure 9 and Figure 10).

## 4 Discussion

### 4.1 Summary of results

In this study, based on lump-parameter Windkessel models with age-dependent non-linear elements, we simulated the effect of ICP increase due to intracranial capacitance decrease on the waveform features of PPG signals of different cerebral perfusion territories in subjects of different ages. The simulation results showed that ICP changes could significantly influence the maximum, minimum, and amplitude of PPG signals, with limited effect on min-to-max time, PI, RI, and MMR. As far as we know, this is the first study that quantitatively investigates the effect of ICP on the waveform features of intracranial PPG signals using computational simulation.

### 4.2 Clinical need on non-invasive ICP monitoring: A wearable pathway *via* PPG?

Recent years have witnessed the development of non-invasive ICP monitoring technologies, including transcranial Doppler measurement of cerebral blood flow, near-infrared spectroscopy (NIRS), tympanic membrane displacement (TMD) (Lee et al., 2020), ophthalmodynamometry (Nag et al., 2019), optic nerve sheath diameter (ONSD) analysis based on ultrasound (i.e., transcranial Doppler) or radiological [e.g., computed tomography (CT), MRI, and optical coherence tomography (OCT)] data, and other imaging-based methods (e.g., analysis of CT-derived ratio of CSF volume to the total intracranial volume) (Harary et al., 2018; Nag et al., 2019). These techniques enable the non-invasive measurement of ICP in clinical practice. However, these methods depend on expensive devices or clinical imaging data, which require professional skills of operation and data processing. Considering the risk of infection and limited medical resources, the postoperative ICP monitoring was often performed for a couple of days or a week for invasive and non-invasive methods, respectively, despite its clinical significance (Chang et al., 2019; Chang et al., 2021). To achieve better postoperative management of TBI patients, there is a high clinical need for easy-to-perform and low-cost techniques of non-invasive long-term ICP monitoring.

Compared with existing techniques, PPG signals can be detected from different body sites using low-cost wearable sensors without any need for expertise or training. PPG technology has been widely used in healthcare monitoring and early detection of cardiovascular diseases (Allen, 2007; Liu et al., 2019). Transcranial Doppler ultrasonography (TCD) and servo-controlled finger PPG have been applied in continuous bedside monitoring of cerebral blood flow and blood pressure, as well as the evaluation of cerebral autoregulation (Aries et al., 2010). Some pilot studies showed that PPG waveform features may indicate pathological hemodynamic changes in cerebral circulation on which ICP has a strong influence. The amplitude of PPG signals measured from bilateral index fingers is associated with cerebral artery stenosis (Kang et al., 2018). The TCD-derived PI and PPG waveform are associated with cerebrovascular hemodynamic changes in the patients with the disorder of consciousness (Liu et al., 2016). Morgan et al. (2014) developed a modified PPG system using video recordings taken through an ophthalmodynamometer and timed to the cardiac cycle to investigate the phase lag between retinal venous and arterial pulses. Based on this modified PPG system, Abdul-Rahman et al. (2020) recently estimated ICP value from retinal vascular pulse wave attenuation. Abnormal morphological and biomechanical properties of retinal veins have been proven to be biomarkers to guide diagnosis and management of elevated ICP (Moss, 2021). In accordance with existing studies, our results provided new evidence that waveform features (i.e., maximum, minimum, mean, and amplitude) of intracranial PPG signals could reflect the changes in ICP. Therefore, PPG technology may enable the non-invasive long-term ICP monitoring.

Meanwhile, the majority of existing studies on PPG-assisted ICP monitoring are based on the PPG signals of fingers, retina, or other extracranial sites. The transcranial brain PPG technology was developed to study the venules of cerebral cortex (Viola et al., 2013) but has not been applied in ICP monitoring. Our results provide new reference on ICP estimation based on intracranial PPG signals which directly reflect the status of cerebral microcirculation.

### 4.3 ICP-relevant PPG waveform features

We observed that ICP significantly influenced the value-related waveform features (i.e., maximum, minimum, mean, and amplitude), with negligible effect on shape-related ones (i.e., min-to-max time, PI, RI, and MMR). Especially, the ICP changes did not generate any consistent differences in PI, which is in accordance with Fernando et al.‘s observation in a recent systematic review that PI derived from TCD signal (TCD-PI) has poor accuracy in estimating ICP (range of area under the receiver operating characteristic curve: .550–.718) (Fernando et al., 2019). Here we try to provide an initial explanation on this phenomenon from a physiological perspective based on our computational model. The changes in intracranial capacitance influence the ICP, thus the boundary conditions of intracranial arteries in the cardiocerebral artery network. However, the fluctuations of ICP signals in a cardiac cycle are limited (amplitude<5 mmHg, Figure 6 and Figure 8). Thus, the increase of ICP changes the value of outlet pressure in the model, without generating much pulsatility at the outlets. On the other hand, ICP is much lower in value than the blood pressure, which does not change the biomechanical properties of the vessel wall on both macro- and microvascular levels. Therefore, ICP can significantly change the value-related PPG waveform features of ICP with minor effect on the shape-related ones.

### 4.4 Role of other physiological factors in ICP-induced PPG waveform changes

We observed strong effects of age and measurement site (i.e., cerebral perfusion territory) on intracranial PPG waveform features. The PPG waveform depends on many physiological features including age, measurement site, blood pressure, respiratory pattern, and neural activities (Liu et al., 2020a). The biomechanical properties of the cardiovascular system (e.g., arterial stiffness) depends on the age. In Figure 9, age-related changes in PPG waveform features are more significant than ICP-related ones. Age-adjusted analysis can be considered in PPG-based ICP estimation. However, the effect of age on the mean is negligible, which indicates that the normal intensity of cerebral microcirculation is unaffected by age (Cidis Meltzer et al., 2000).

PPG waveform also strongly depends on the blood pressure value and can be used for blood pressure estimation (Allen, 2007). The combination of TCD and blood pressure showed much higher accuracy than the TCD-PI method in estimating ICP (Fernando et al., 2019). Ruesch et al. (2020) investigated the estimation of ICP based on cerebral blood flow measured by diffuse correlation spectroscopy, and found an obvious improvement in accuracy when mean arterial blood pressure was included (R-squared values: .82 and .92). Furthermore, the ICP-induced dysfunction of cardiorespiratory system and cerebral autoregulation can lead to complex changes in cerebral microcirculation and resultant PPG waveform (Winklewski et al., 2019). Therefore, other physiological factors and their interactions deserve further consideration in investigating the relationship between ICP and PPG waveform features.

### 4.5 Towards better accuracy: Individualization of arterial parameters and venous model

**FIGURE 11:**
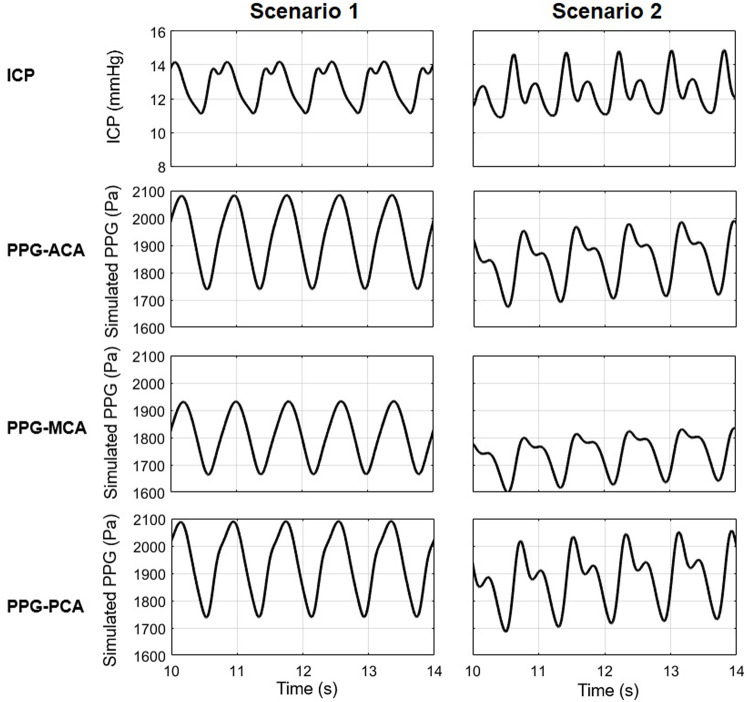
The ICP and PPG signals simulated in two scenarios with different values of the elements in cardiocerebral artery network.

The proposed model consists of 33 artery segments from aorta to the Circle of Willis. To generate reliable simulation results for clinical application, the biomechanical properties of the arteries need to be evaluated individually in different subjects. In this model, the biomechanical and anatomic properties of the arteries were derived from some earlier physiological measurement results (Stergiopulos et al., 1992; Fahrig et al., 1999; Moore et al., 2006) where the properties distributed in wide ranges. We noted that the parameters of vascular anatomy in Alastruey et al. (2007) and Zhang et al. (2014) models were not exactly the same. All the values fell in the normal ranges, whilst the differences in anatomic parameters provided a chance to observe the effect of individual vascular anatomy on the simulated ICP and PPG signals. Figure 11 shows the simulation results of a 40-year old male subject with 25% decrease in intracranial capacitance based on Alastruey et al. (2007) and Zhang et al. (2014) arterial models (scenarios 1 and 2, respectively), with the parameters of other parts identical. The ICPs of both scenarios are similar in range but different in waveform. The PPG signals of both scenarios are different in range and waveform, whereas, similar trends can be observed, i.e., the PPG of MCA territory is lower in amplitude, maximum, and minimum compared with those of ACA and PCA territories. Therefore, this model initially indicated the possibility of PPG-based ICP estimation, while there is a long way to explore towards individualization of the model where patient-specific anatomic data are essential.

In addition, the simulated ICP has one or two peaks, while *in vivo* ICP often has three peaks in a cardiac cycle: P1 (percussion wave), P2 (tidal wave), and P3 (dicrotic wave) (Harary et al., 2018). This might partly due to the simplification of venous circulation and its interaction with ICP. Although the precise origin of ICP peaks is not fully understood yet, P2 and P3 are often thought relevant to the retrograde venous pulse of the jugular against the cortical veins (Rodríguez-Boto et al., 2015). In the classic ICP model which we adopted, the cerebral venous system was simplified as a unilateral flow dependent on ICP (Ursino and Di Giammarco, 1991; Lee et al., 2015). Some advanced mathematical models have been proposed to describe the non-linear hemodynamic properties of cerebral veins (Toro, 2016). However, these models have not been fully validated on patients with different ICP levels. Considering the complexity and individual difference of cerebral venous system, patient-specific hemodynamic data (e.g., MRI-derived flow) are essential in the individualization of the cerebral circulation model measurement (Müller and Toro, 2014). A computationally efficient model that reflects the interaction between ICP and intracranial venous system is essential for improving the accuracy of ICP waveform estimation.

### 4.6 Limitations and future directions

There are some limitations in this pilot study. First, as aforementioned, the model was an idealized one where the values of elements and boundary conditions were derived from literature. Considering the individual difference in waveform which may involve other confounders, we did not include the analysis of focal waveform features, e.g., the location of maximal/minimal first or second derivatives. These features may reflect important physiological information including neural activities (Khalid et al., 2022) and cardiovascular pathophysiological changes (Elgendi et al., 2018). Second, the Windkessel models were highly simplified where the local hemodynamic changes within an arterial segment or a perfusion territory could not be reflected. For simplification, the aging effect was only considered in aorta and big arteries. The aging effects on cerebral vasculature (Oudegeest-Sander et al., 2014; Graff et al., 2021) and veins (Fulop et al., 2019; Huang et al., 2021) were not included in the proposed model due to the lack of comprehensive measurement results among subjects with different ages and ICP levels. Cerebral autoregulation and respiration can also significantly influence the dynamics of ICP (Budohoski et al., 2012; Vinje et al., 2019). In addition, the ICP model was simplified as a unidirectional flow system where the interactions between cerebral ventricles were not included. The PPG signals was also highly simplified as the pressure drop due to volumetric changes. The optical and electronic components were not included. In real-world scenarios, the PPG signals are sensitive to many physiological and technical factors, e.g., motion artefact, contact pressure, *etc.*, which can significantly deform the PPG signals (Fine et al., 2021). It needs further validation whether the ICP-related changes can be reliably detected from the real-world noisy PPG signals. Most importantly, the ICP values were generated by setting different intracranial capacitance decrease levels, while an elevated ICP could be generated by different pathological mechanisms where multiple physiological factors are involved.

In future studies, by introducing patient-specific biomechanical parameters and hemodynamic parameters as boundary conditions, using more advanced biomechanical models (e.g., venous valves, starling resistors) especially in cerebral venous system (Toro et al., 2022), adding optical sensing components, and including more physiological factors (e.g., respiratory regulation), the relationship between ICP and the waveform features of intracranial PPG signal could be further investigated in different pathological conditions.

## 5 Conclusion

ICP values could significantly change the value-relevant (maximum, minimum, mean, and amplitude) waveform features of PPG signals measured from different cerebral perfusion territories, with negligible effect on shape-relevant features (min-to-max time, PI, RI, and MMR). In addition, age and measurement site significantly influence all PPG waveform features except the mean.

## Data Availability

The original contributions presented in the study are included in the article/Supplementary Material, further inquiries can be directed to the corresponding authors.
